# Book Reviews

**Published:** 1892-11

**Authors:** 


### Book Reviews.

AN AMERICAN TEXT-BOOK OF SURGERY FOR PRACTITIONERS AND STUDENTS. By Charles H. Burnett, M. D., Phineas S. Conner, M. D., Frederic S. Dennis, M. D., William W. Keen, M. D., Charles B. Nancrede, M. D., Roswell Park, M. D., Lewis S. Pilcher, M. D., Nicholas Senn, M. D., Francis J. Shepherd, M. D., Lewis A. Stimson, M. D., William Thomson, M. D., J. Collins Warren, M. D., and J. William White, M. D. Edited by William W. Keen, M. D., LL.D., and J. William White, M. D., Ph. D. Profusely illustrated. Price, $7.00 net, cloth ; $8.00, sheep ; $9.00, half Russia. Sold by subscription only. Philadelphia : W. B. Saunders, 913 Walnut street. 1892.

The tendency now is towards encyclopedic medical literature. In this work a number of distinguished teachers and practitioners appear as authors, but it is impossible, except by inference, to differentiate the part that each has taken in writing the book. The scope and plan is somewhat novel, but in a sense appeals to the conditions that confront us. Beginning with Surgical Bacteriology, which occupies the first chapter of ten pages, the subjects are taken up in their regular order. In book first they follow that of treatises upon general surgery.

Book second is devoted to Special Surgery, and has ten chapters, varying from ten to thirty pages each. The classification by anatomical systems is an admirable one. Chapter I. deals with Surgery of the Vascular System; Chapter II. with that of the Oseous System; Chapter III. with Fractures; Chapter IV. with Diseases and Injuries of Muscles, Tendons, and Bursæ; Chapter V. with Orthopedic Surgery; Chapter VI. with Surgery of the Nerves; Chapter VII. with Surgery of the Joints; Chapter VIII. with Dislocations; Chapter IX. with Injuries of the Lymphatics; Chapter X. with Surgery of the Skin and Appendages. We have enumerated the subdivisions of book second for the purpose of calling attention to the admirable classification which the editors have made.

Book third deals with Regional Surgery, beginning with Diseases and Injuries of the Head, and going through the several regions, cavities, and tracts of the body, including Surgery of the Eye and Ear, besides devoting a chapter each to the surgery of the genito-urinary tracts of the male and female. Book fourth is devoted exclusively to operative surgery, while a voluminous index of twenty-two pages closes the volume.

There are some admirable features about this work, and we have no doubt it will assume a popular place in the literature of surgery and be found prominently displayed on the book-shelves of surgeons, or even of those who have an occasional necessity to practise that art. We are of the opinion that it would have been well to have omitted such chapters as the Surgery of the Female Generative Organs, or else have given the subject over to the hands of an experienced gynecologist to prepare. The general surgeon rarely writes anything that attracts the specialist when he deals with this department, and, we may say, still more rarely writes anything useful to the student. Such chapters are generally padded with a few glittering generalities, together with a number of indifferent illustrations that are either obsolete or else so painfully fresh as to have little or no practical utility. This criticism may also be fairly offered with regard to the other special departments of surgery, such as the eye and ear.

This work is admirably printed on beautiful paper, and some of the plates are especially fine. Many of the cuts have, heretofore, done long duty and are now veterans, while a large number are original, and will command careful inspection. Facing page 896 we find plate 22, "hypertrophy of the median and lobes of the prostate," has been reversed, so that it reads upside down, and there are some few further slight defects due to hasty preparation, but these are of small moment. We have no patience, however, with hypercritical remarks regarding the mechanics of a book when the text is in the main so admirable. The book is rather cumbersome to handle, and needs a very strong binding, which, we are sorry to say, the one we possess has not. The authors have been admirably grouped with reference to their special fitness to write practical surgery, and the editors could not have been more wisely chosen. On the whole, we commend this volume as the most interesting and instructive American text-book contribution to the literature of surgery made by American surgeons within the last quarter of a century.

---

A TREATISE ON DISEASES OF THE NOSE AND THROAT, in two volumes. By FRANCKE HUNTINGTON BOSWORTH, A. M., M D., Professor of Diseases of the Throat in the Bellevue Hospital Medical College, New York ; Consulting Laryngologist to the Presbyterian Hospital; Consulting Physician to the O. D. P. of the Bellevue Hospital ; Fellow of the American Laryngological Association, of the American Climatological Association, of the New York Academy of Medicine ; Member of the New York Laryngological Society, of the Medical Society of the County of New York, etc., etc. Volume II. Diseases of the Throat. With three colored plates and 125 wood-cuts. New York : William Wood & Co. 1892.

The continuation of the consideration of this subject by Bosworth will be a source of gratification to every member of the profession who is interested in the study of the diseases of which it treats. The first volume of this important work appeared about three years ago, and received an extended notice in Vol. XXIX., page 449, of the JOURNAL, February, 1890. The volume before us is divided into three sections, the first of which deals with Diseases of the Fauces. Chapters I. and II. are devoted to the Anatomy and Physiology of the Fauces, and are a concise, but minute statement of these subjects. Acute and Chronic Follicular Pharyngitis are dealt with in the next three chapters, and throw much light on a subject that is, perhaps, the most obscure or least attractive of any questions connected with laryngology. Retro-pharyngeal abscess is an important disease, one singularly fatal, one not always easily recognized, and either leading to systemic or fatal local complications promptly. Bosworth handles it with directness, and upon surgical principles. The uvula and tonsils come in for their share of appropriate treatment, and in Chapter XVI. diphtheria is dealt with in sixty pages of text. Bosworth holds to the doctrine of Oertel that diphtheria is primarily a local disease, which we regard as the only sound view of its pathology to take in the light of our present knowledge. But Bosworth, like all other advanced thinkers, recognizes the rapidity with which systemic invasion of the poison may follow its local appearance, and, hence, is not slow to treat it accordingly. We are pleased to observe the emphasis which he places upon the proper examination of the fauces of a diphtheritic patient. "It is not sufficient," he says, "to examine the child's throat by means of a candle, held in front of the face; the illumination should always be accomplished, where feasible, by means of a light reflected into the fauces from the concave mirror, while, at the same time, the parts are thoroughly exposed by the deft manipulation of a tongue depressor, and not a tablespoon, or such other domestic instrument as may be at hand. I do not think these points can be insisted upon too strongly." In making this examination, Bosworth recognizes its dangers and advises that the exposed parts of the operator, namely, the mouth and eyes, be protected, the former by a handkerchief and the latter by eye-glasses. If physicians would pay more attention to this necessary protection, we should hear of less sacrifice of life among those who attend diphtheritic patients. Chapter XVII. is an able exposition of our present knowledge with reference to Syphilis of the Fauces. Then comes, in succession, chapters on Tuberculosis of the Fauces, Lupus of the Fauces, and Foreign Bodies in the Fauces. Neuroses, Tumors, Sarcoma, and Carcinoma of the Fauces follow each other in consecutive order. Section II. deals with Diseases of the Larynx, and follows much the same order of sequence in which the diseases of the fauces were considered in Section I. We need not take time now to speak of all the details of this section, but it must suffice for us to say that to read it is to be instructed in a high degree.

Finally, the volume closes with the consideration, in Section III., of the External Surgery of the Throat. Minute and careful details are given for the operations of laryngotomy, thyrotomy, tracheotomy, extirpation, and resection of the larynx. The book is most handsomely printed on clear paper and plain type, and voluminous references are given at the foot of almost every page. A comprehensive biographical and general index makes it useful for reference. In conclusion, we unhesitatingly pronounce Bosworth's work, in the two volumes, altogether the most useful textbook contribution to the knowledge of disease of the nose and throat that has yet been handed out to a progressive and intelligent profession.

---

Book on the Physician Himself, and Things that Concern His Reputation and Success. By D. W. Cathell, M. D., Baltimore, Md. New tenth edition (author's last revision). Thoroughly revised, enlarged, and rewritten. In one handsome royal octavo volume. 348 pages. Bound in extra cloth. Price, post-paid, $2.00 net. Philadelphia: The F. A. Davis Co., Publishers, 1231 Filbert street.

At first glance, it is not easy to see what useful end was to be served by the publication of this remarkable book, and it is still more difficult to account for the popularity it has gained in view of the fact that the volume before us is a copy of the tenth edition. No one would charge Dr. Cathell with a wish to bring the medical profession into disrepute, and yet the unprofessional reader, especially if he be one of the many who are ready to belittle the value of medical science, will not be likely to have his distrust of a profession removed whose members need to be instructed in, or even reminded of, the most ordinary and obvious rules of ethics, not to mention the numerous professional directions, which, by implication, charge the practitioners, for whom the book is supposed to be written, with carelessness or ignorance.

To educated physicians the advice given by Dr. Cathell is, to say the least, gratuitous, since they will see in his best recommendations simply a mass of truisms; while upon those whose ignorance or moral twist ought to have prevented their investiture with the right to practise medicine, the really valuable part of the work is probably thrown away. Some of these, however, will appreciate the lesson given in behalf of personal advantages. Take one example: After recommending a familiarity with general scientific subjects and general knowledge in order to be on "a conversational level" with cultured classes with whom one may be brought into contact, he goes on to clinch his subject, so to speak, with the following worldly wisdom: "In fact, among intellectual and educated people, good conversational powers and broad culture, often, actually produce a higher opinion of a physician's professional ability than is really possessed."

One plausible reason for the original appearance of the book, a reason happily growing less and less valid, might be urged, viz., the ease with which a diploma could be had in some sections of the United States, has flooded the country with a mass of uneducated doctors, most of whom had entered the profession simply for gain. Some there were, however, who were anxious to supply the deficiencies of early education by diligent and earnest study. To such, some of the advice given must have been of value. Others, and these were probably in the majority, cared little for science, so long as business prospered, and would, therefore, receive little benefit, except from such parts as treat of matters for personal advantage.

Since the first issue of Dr. Cathell's book, the times have changed in regard to the science and practice of the healing art. While this change is gradually going on all over our country, it is felt less in some parts than others, though there is room to hope that, by the united exertions and influence of the regular medical societies, a consensus of opinion will be formed, at no distant period, which will compel a high standard in all the schools of medicine, making an ignorant physician an anomaly if not an impossibility. We are proud to say that, in our own section of the country, this hope is realizing to a degree unthought of a quarter of a century ago, and that, by the concentrated efforts of the Medical Society of the State of New York, the laws of the State have been successfully invoked to prevent frauds upon the people by spurious or purchased diplomas, making them no longer the æges behind which they can conceal their ignorance.

---

A Text-Book of the Principles and Practice of Medicine. For the use of students and practitioners, By Henry M. Lyman, M. D., Professor of the Principles and Practice of Medicine in Rush Medical College, Chicago. In one very handsome octavo volume of 926 pages, with 170 illustrations. Cloth, \$4.75 ; leather, \$5.75. Philadelphia : Lea Brothers & Co. 1892.

Text-books on the practice of medicine are multiplying with wonderful rapidity. The reviewer has hardly laid aside Osler's recent remarkable treatise before he is called upon to go through the same process with reference to this sturdy candidate for favor and patronage that comes from the West. Lyman says he has endeavored to present, in this book, not only the fruits of his own observations and experience, but, in substance, the latest editions of the works of Ziegler, Hallopeau, Eichhorst, Cornil, and Babes, and of the collaborators of the Traité de Médecine.

Lyman divides his book into thirteen parts, and has departed from the usual order of sequence in the arrangement of topics.

Part I. is taken up with the consideration of preliminary matters, such as Growth and Development, Disturbances of Nutrition, Tumors, Disorders of Circulation, Contagion, Inflammation, and Fever. In Part II. he considers Parasitic Infective Diseases. Much interest will naturally center in any consideration of influenza or grippe, but Lyman devotes only a short chapter to this important subject. It consists of only four and a half pages of reading matter. The directness in style of the author, however, enables him to condense into these four and a half pages considererable valuable information. But, for a thorough understanding of this recently revived epidemic infective disease, it will be necessary to consult more elaborate treatises; or, still better, a monograph especially prepared by a competent man who shall treat the subject exhaustively. In Part III. we have a consideration of the Diseases of the Alimentary Canal, and here we find nothing beyond the usual order in handling the subject, though Lyman is precise in its treatment. He begins with diseases of the mouth, and considers every subdivision of the alimentary canal downward, until the lower intestine is reached, classifying each subdivision with reference to etiology, symptoms, diagnosis, prognosis, and treatment, together with such brief pathological considerations as the scope of his treatise will permit.

In Part IV., Diseases of the Liver, Pancreas, Peritoneum, and Spleen are considered. We think it would be well if most physicians, especially those who are apt to find their patients "bilious," or with their livers out of order, to read every author who has lately written on the diseases of that much-abused, but still very useful, organ. We remember that it has been said by one of the distinguished teachers of the land, that whenever we hear a doctor talk about liver complaint and biliousness, this should be set down as an expression of the algebraic formula of the unknown quantity of that man's brain. Let us, therefore, hear less of "biliousness" and "liver complaint," and come down to particulars when discussing or treating disturbances of the digestive tract, that are supposed to arise from functional or other faults of the liver. This is the line on which Lyman handles the subject.

Part V. treats of the Diseases of the Organs of Respiration, and this will be considered by many the most interesting section of the book, as it shows much care and exactitude in its preparation. The same, too, may be said of Part VI., which considers Diseases of the Organs of Circulation, to which 100 pages are devoted.

The remaining sections we must pass, with the final remark that the treatise is made up in a way to command the attention of the student, who must necessarily consult books that are concisely written, for under the pressure of time, which is too short for his proper preparation, he must necessarily submit to the baneful influences of the cramming process, that so implants itself upon his brain during his early student years, as to seldom or never become fully eradicated in after life. The author of this work is a man of distinction, and holds a teacher's chair in one of the most prominent medical schools in the West; therefore, on account of his scholarly attainment and high position, will command the respect of, and hearing from, a large and intelligent audience. We commend his labor as having been conscientiously performed, and as being entitled to an equally conscientious and patient reading. This treatise is, beyond all others issued of late, the one for a student to purchase and adopt as his guide.

---

OUTLINES OF ZOOLOGY. By J. ARTHUR THOMSON, M. A., F. R. S. E., Lecturer on Zoology in the School of Medicine, Edinburgh; joint-author of the Evolution of Sex; author of the Study of Animal Life. With thirty-two full-page illustrations; small 8vo, pp. 641. New York: D. Appleton & Co. 1892.

Books, though they cannot make one a naturalist without practical observation, are, in the present state of science, indispensable in directing his studies. The volume before us appears to be one of utmost importance in this direction, not only to the beginner, who must find in its pages a wealth of information that years of observation alone would fail to give him, but even to the advanced student of zoology, to whom it will serve as a reference where the essentials of the science are grouped together and stated in form and manner so clear and concise that he can at a glance revive his memory and fix his data.

Although Mr. Thomson modestly calls his production an "outline," it is far from being like some works on scientific subjects, a mere arrangement of orders, genera and species, by which the student may determine to what class his specimen belongs. From the amorphous protoplasm we may here trace the evolution of the various forms of animal life, from the simple amœba to the most complex vertebrate. The chapters on Evolution, Heredity, and Types will be found particularly interesting, and nothing can be more so than the clear and beautiful descriptions of the anatomy, physiology, and habits of some of the lower animals, whose lives and habitats render their observation and description very difficult.

Excellent wood-cuts illustrate the whole, and the typography is of the best, making the volume one of exceptional value and interest to the student.

---

Essentials of Medical Diagnosis, arranged in the form of questions and answers, prepared especially for students of medicine. By Solomon Solis-Cohen, M. D., Professor of Clinical Medicine and Applied Therapeutics in the Philadelphia Polyclinic; one of the Physicians to the Philadelphia Hospital, etc.; and Augustus A. Eshner, M. D., Instructor in Clinical Medicine in Jefferson Medical College and in the Philadelphia Polyclinic; Registrar in the Neurological Deparment of the Philadelphia Hospital, etc. With fifty-five illustrations, some of which are colored, and a frontispiece. Philadelphia: W. B. Saunders, 913 Walnut street. 1892.

This book, which is number seventeen of the Essentials series, is more elaborate than its predecessors, and may be very properly so constructed, for it lays a foundation for future success to become a good diagnostician. This book has been, like its fellows, written especially for students, and to them it must look for its patronage. Since teachers themselves are responsible for the demand that is made for this form of book, it is not worth while to criticise the students if they choose to purchase it. It certainly will facilitate the cramming process, which is so necessary for a final examination, where the years allotted to student life are so few and the terms so short. We commend this book as one of the best of its kind.

---

A System of Gynecology, with 359 illustrations; based upon a translation from the French of Samuel Pozzi. Revised by Curtis M. Beebe, M. D., Chicago, Ill. New York: J. B. Flint & Co.

This abridgment of Pozzi's great work appears to have been issued for the purpose of economizing the cost, and further to give its editor a prominence. The authorized edition, edited by Dr. Brooks H. Wells, and published by William Wood & Co., is, however, the one that the profession will naturally choose, and it is certainly the one that ought to command its attention and patronage. The edition before us, although printed on good paper and with good type, is so condensed that it conveys only a meager impression of Pozzi's original work. We cannot consent to record our approba tion of this method of reproducing an author's elaborate and exhaustive treatise and work, that has been the pride of his life and cost him many years of study and preparation.

---

DELSARTEAN PHYSICAL CULTURE, with Principles of the Universal Formula. By CARRICA LE FARRE. New York: Fowler & Wells Co., Publishers, 775 Broadway. 1891.

This little book is invaluable to the student of physical culture. It sets forth the principles of physical culture in a comprehensive and practical manner. It enables anyone to become thoroughly acquainted with the system without belonging to a class. The author understands her subject thoroughly.

